# Lipoxins and Resolvins in Patients With Pancreatic Cancer: A Preliminary Report

**DOI:** 10.3389/fonc.2021.757073

**Published:** 2022-01-11

**Authors:** Wojciech Blogowski, Katarzyna Dolegowska, Anna Deskur, Barbara Dolegowska, Teresa Starzynska

**Affiliations:** ^1^ Institute of Medical Sciences, University of Zielona Gora, Zielona Gora, Poland; ^2^ Department of Microbiology, Immunology and Laboratory Medicine, Pomeranian Medical University, Szczecin, Poland; ^3^ Department of Gastroenterology, Pomeranian Medical University, Szczecin, Poland

**Keywords:** immunoresolvents, lipoxygenase, lipoxin, pancreatic cancer, resolvin

## Abstract

Eicosanoids are bioactive lipids derived from arachidonic acid, which have emerged as key regulators of a wide variety of pathophysiological processes in recent times and are implicated as mediators of gastrointestinal cancer. In this study, we investigated the systemic levels of lipoxygenase (LOX)-derived lipoxin A4 and B4, together with resolvin D1 and D2 in patients with pancreatic adenocarcinoma (n = 68), as well as in healthy individuals (n = 32). Systemic concentrations of the aforementioned immunoresolvents were measured using an enzyme-linked immunosorbent assay (ELISA). In this study, we observed that compared with concentrations in healthy individuals, the peripheral concentrations of the aforementioned eicosanoids were significantly elevated (2- to 10-fold) in patients with pancreatic cancer (in all cases p<0.00001). No significant association was observed between eicosanoid levels and the TNM clinical staging. Furthermore, we observed no significant differences in concentrations of the analyzed bioactive lipids between patients diagnosed with early-stage (TNM stage I-II) and more advanced disease (TNM stage III-IV). Receiver operating characteristic (ROC) curve analysis of each aforementioned immunoresolvent showed area under the curve values ranging between 0.79 and 1.00. Sensitivity, specificity, as well as positive and negative predictive values of the eicosanoids involved in the detection/differentiation of pancreatic adenocarcinoma ranged between 56.8% and 100%. In summary, our research is the first study that provides clinical evidence to support a systemic imbalance in LOX-derived lipoxins and resolvins as the mechanism underlying the pathogenesis of pancreatic adenocarcinoma. This phenomenon occurs regardless of the clinical TNM stage of the disease. Furthermore, our study is the first to preliminarily highlight the role of peripheral levels of immunoresolvents, particularly resolvin D1, as potential novel biomarkers of pancreatic cancer in humans.

## Introduction

Pancreatic adenocarcinoma is an extremely aggressive and invariably fatal malignancy in humans. Approximately 60,000 individuals are diagnosed with this malignancy that is known to cause 50,000 deaths annually in the United States. Multiple risk factors including age, certain genetic syndromes, smoking, diabetes, alcohol abuse, and obesity are implicated as etiopathogenetic contributors to pancreatic adenocarcinoma, and several molecular pathways associated with pancreatic cancer have been identified (1, 2). However, the exact mechanisms underlying this disease remain unclear. Therefore, prevention, early detection, and prompt treatment are clinically challenging.

Bioactive lipids derived from arachidonic acid (AA), referred to as eicosanoids are implicated as key factors in carcinogenesis, in recent times. These AA derivatives represent a large family of substances that affect multiorgan function, including gastrointestinal physiology and regulate several pathophysiological processes in the body, such as vascular flow, angiogenesis, cellular proliferation, inflammation, and metabolism (3–6). From the biochemical viewpoint, AA-derived eicosanoids are produced *via* the CYP450, cyclo-oxygenase (COX), or lipoxygenase (LOX) enzymatic pathways (7, 8). Among the various eicosanoids generated *via* the aforementioned enzymatic pathways, bioactive lipids such as leukotrienes, hydroxyeicosatetraenoic acids, lipoxins, and resolvins have gained much attention as significant contributors to malignancies. This effect is mainly associated with their actions on immune cell function and modulation of both initiation (leukotrienes) and resolution (lipoxins, resolvins) of inflammatory processes (9–12). Although acute inflammation is usually a physiological response that protects the body from temporary microbial infections or injurious stimuli, uncontrolled chronic inflammation predisposes to carcinogenesis *via* DNA injury, epigenetic dysregulation, genomic instability, and/or changes in intracellular signaling. LOX-derived lipoxins and resolvins participate in resolution of inflammation; several experimental studies have shown their benefits in suppression of chronic inflammation-induced tumorigenesis (13, 14). These results support the potential application of these immunoresolvents as promising preventive or anti-cancer agents (15–18); however, limited information is available regarding their role in the development of pancreatic adenocarcinoma in humans.

In this study, we preliminarily investigated the systemic concentrations of lipoxins (A4 and B4) and resolvins (D1 and D2) in patients with pancreatic cancer and compared these values with those observed in healthy individuals. Moreover, we investigated the association between clinical TNM staging of pancreatic adenocarcinoma and immunoresolvents’ levels in patients with cancer. We additionally investigated whether systemic concentrations of lipoxins and resolvins show any significant diagnostic value for detection/differentiation of pancreatic cancer.

## Material and Methods

### Study Participants and Clinical Protocols

We recruited 100 individuals in this study. All participants were evaluated (in an outpatient or inpatient setting) at our Department and were confirmed to be stable with good general health. Exclusion criteria were as follows: an active infectious/inflammatory disease, a history of any malignancy, administration of medications that could potentially interfere with AA metabolism (such as COX inhibitors), a history of blood transfusions within 6 months prior to study enrollment, active supplementation of lipid derivatives (omega-3 fatty acids), and/or refusal to participate in the study.

Among the 100 patients recruited for the study, 68 were diagnosed with pancreatic adenocarcinoma and were categorized into the “cancer” group. Similar to previous studies performed by our group (19–21), diagnosis of pancreatic cancer was based on evaluation of biopsy specimens obtained *via* endoscopic ultrasound, paracentesis (in patients with neoplastic ascites), or liver biopsy (in patients with metastatic disease). All patients underwent laboratory tests and imaging (abdominal/chest computed tomography and/or abdominal ultrasonography), and these results were subsequently used for TNM staging of the cancer. In this study, stage I and II pancreatic adenocarcinoma was diagnosed in 5 and 17 patients, respectively. Advanced-stage disease (stage III) occurred in 11 patients and metastatic disease (stage IV) in 35 patients. All patients had a recent diagnosis of pancreatic cancer at the time of study inclusion; therefore, no patient received any chemotherapy or any cytotoxic therapy within a year preceding the diagnosis, and no active acute infection or disease was observed in any patient.

The control group in our study included 32 volunteers in an overall good state of health.

### Blood Sample Collection and Systemic Immunoresolvent Level Measurements

Peripheral blood samples (8–10 mL) were obtained from all individuals enrolled in this study. The samples were immediately processed based on standard laboratory protocols; plasma was separated, frozen, and stored at –80°C until further tests were performed. The systemic concentrations of analyzed immunoresolvents (lipoxin A4 and B4 and resolvin D1 and D2) were measured using commercially available, high-sensitivity enzyme-linked immunosorbent assay kits (*Wuhan EIAab Science Co, Ltd.*, China and *Cayman Chemicals*, MI, USA) based on the manufacturers’ instructions.

### Statistical Analysis

Similarly to our previous studies (22, 23) all of the received results were subjected to a comprehensive analysis with use of statistical software. Specifically, normality of distribution of the variables was tested using the Shapiro–Wilk test. Continuous variables that were abnormally distributed were subjected to log transformation. Subsequently, if normality of the distribution was obtained then Student’s t-test was used to compare mean values of examined parameters between appropriate groups. Otherwise a Mann–Whitney U-test for non-parametric variables was used. In order to calculate the correlations between parametric and non-parametric variables we used Pearson’s or Spearman’s correlation rank tests (respectively). In addition, a multivariate regression analyses were performed with use of a stepwise selection method. In order to exclude eventual presence of any residual confounding we entered individually the variables that initially were excluded from the constructed model. Finally, we constructed the receiver operating characteristics (ROC) curves and calculated the area under curve (AUC) values for all tested immunoresolvents as eventual diagnostic substances for pancreatic cancer in humans. All of these statistical analyses were performed with use of the SPSS software and p<0.05 values were considered as significant.

## Results

### Baseline Characteristics of Study Participants

**Table 1** summarizes baseline characteristics of the study participants. No statistically significant differences were observed in age and sex distribution, body mass index (BMI), smoking and alcohol consumption habits, and medication history. However, statistical analysis showed significantly higher levels of carbohydrate antigen 19-9 (CA19.9 - pancreatic cancer marker) in patients with pancreatic adenocarcinoma than in healthy controls. Similarly, C-reactive protein levels were significantly higher in the cancer group (**Table 1**).

**Table 1:** General characteristics of analyzed patients and healthy individuals enrolled in the study [data presented as means ± SD or median (interquartile range)].

Parameters	Control	Cancer
Age (years)	61 ± 7	63 ± 11
Gender (M-men/W-women)	14-M/18-W	29-M/39-W
BMI (kg/m^2^)	26.1 ± 4.3	24.0 ± 4.9
Smoking (Y-yes/N-no)	5-Y/27-N	8-Y/60-N
Alcohol (drinks/week)^#^	3.3 ± 1.9	3.4 ± 1.6
Medications (Y-yes/N-no):	25-Y/7-N	55-Y/13-N
Hypertension	25	55
Diabetes	0	0
Lipid lowering (statins)	8	14
Other	1	1
RBC (x10^12^ cells/L)	4.75 ± 0.55	4.31 ± 0.63
Hb (g/dL)	14 ± 1.7	13 ± 1.8
Platelets count (x10^9^ cells/L)	228 ± 60	268 ± 121
WBC count (x10^9^ cells/L)	6.15 ± 1.7	8.6 ± 3.3
CRP (mg/L)	3.0 ± 1.7	27.6 [4.8; 73.1]*
CA19.9 (U/mL)	11.0 ± 5.6	470 [87; 1700]*

BMI, body mass index; RBC, red blood cells; Hb, hemoglobin.

WBC, white blood cells; CRP, C-reactive protein; *P<0.01 (vs “control” group).

^#^a drink was defined as a single consumption of about 8 grams of pure ethanol (equal to for example a glass of wine or a single measure of spirits).

### Peripheral Concentrations of Immunoresolvents in Patients With Pancreatic Cancer vs. Healthy Volunteers

**Figure 1:**
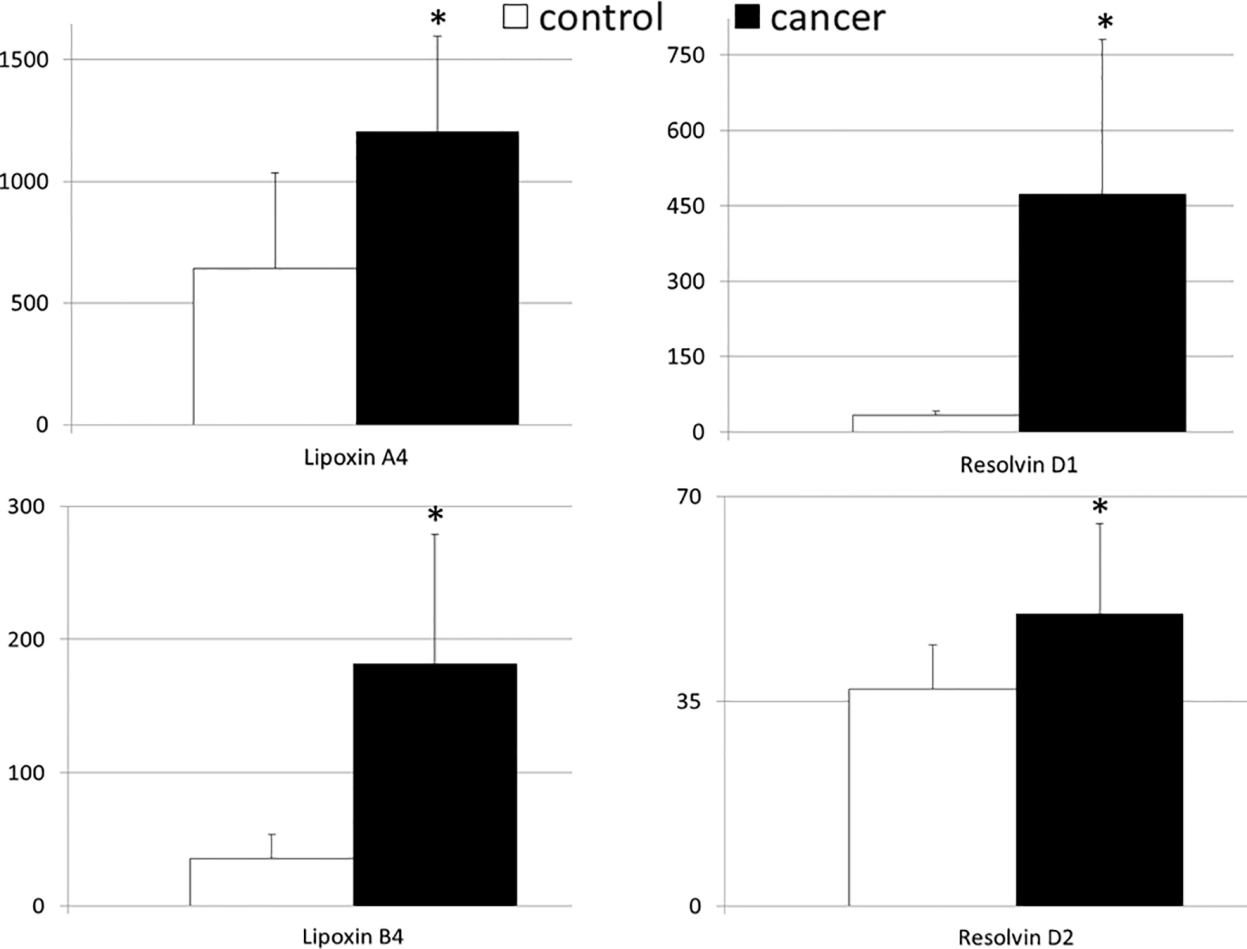
Mean values of the systemic levels of examined immunoresolvents in patients with pancreatic cancer and control individuals together with their statistical comparison (values presented as means ± standard deviation in pg/mL). *p < 0.00001 (vs “control” group).

**Figure 1** shows the mean values of systemic levels of immunoresolvents. We observed that peripheral levels of lipoxin A4 and B4 were significantly higher in patients with pancreatic adenocarcinoma than in healthy individuals (in both cases p<0.00001). Similar findings were observed with regard to resolvin D1 and D2 concentrations. Specifically, the mean peripheral levels of resolvin D1 and D2 were significantly higher (in both cases p<0.00001) in the cancer group than in the control group (**Figure 1**).

### Clinical Association Between Systemic Levels of Immunoresolvents and TNM Staging of Pancreatic Cancer

**Figure 2:**
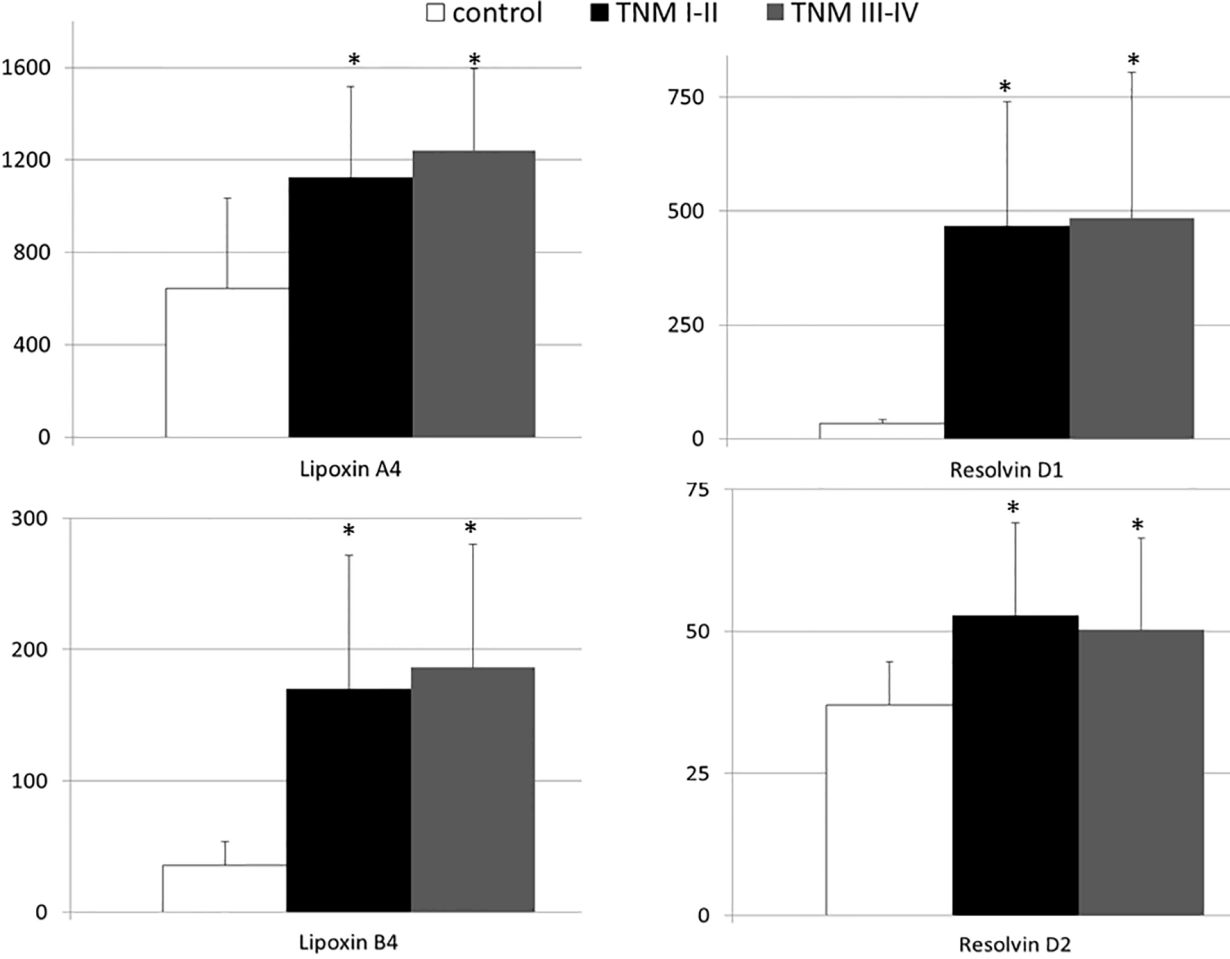
Mean systemic levels of examined immunoresolvents in subgroups of pancreatic cancer patients divided according to the TNM staging and healthy individuals together with their statistical comparison (values presented as means ± standard deviation in pg/mL). *p < 0.003 (vs “control” group).

We investigated the association, if any, between significant changes in systemic levels of immunoresolvents and the clinical staging of pancreatic adenocarcinoma. Correlation analysis showed no significant association between the TNM stage and lipoxin A4 (r=0.08), lipoxin B4 (r=0.13), resolvin D1 (r=0.03), and resolvin D2 (r=0.01) levels (p>0.28 in all cases). Multivariate regression analysis showed similar results (**Table 2**). Additionally, we subcategorized patients from the “cancer” group into two separate subgroups (TNM I–II and TNM III–IV) based on the clinical TNM staging and performed an intergroup comparison of the mean peripheral concentrations of lipoxins and resolvins. Analysis showed no significant differences in mean systemic immunoresolvent levels between patients diagnosed with TNM stage I–II pancreatic cancer and those with more advanced disease (stage III or IV) (p at least >0.21 in all cases) (**Figure 2**).

**Table 2:** Results of statistical analysis of associations between systemic concentrations of examined immunoresolvents and clinical staging of pancreatic cancer in patients (modelling using multivariate regression analysis).

Dependent variable	Independent variable	β	P of the variable	R^2^	P of the model
TNM Staging*	Lipoxin A4	0.08	0.51	0.01	0.51
	Lipoxin B4	0.13	0.28	0.02	0.28
	Resolvin D1	0.03	0.81	0.001	0.81
	Resolvin D2	0.001	0.99	0.001	0.99

β – standardized coefficient in the regression equation; p – level of significance.

*Variable was created by assigning 1, 2, 3 or 4 value to appropriate TNM stage that was present in patients with pancreatic cancer.

### Immunoresolvents as Potential Markers of Pancreatic Adenocarcinoma

**Figure 3:**
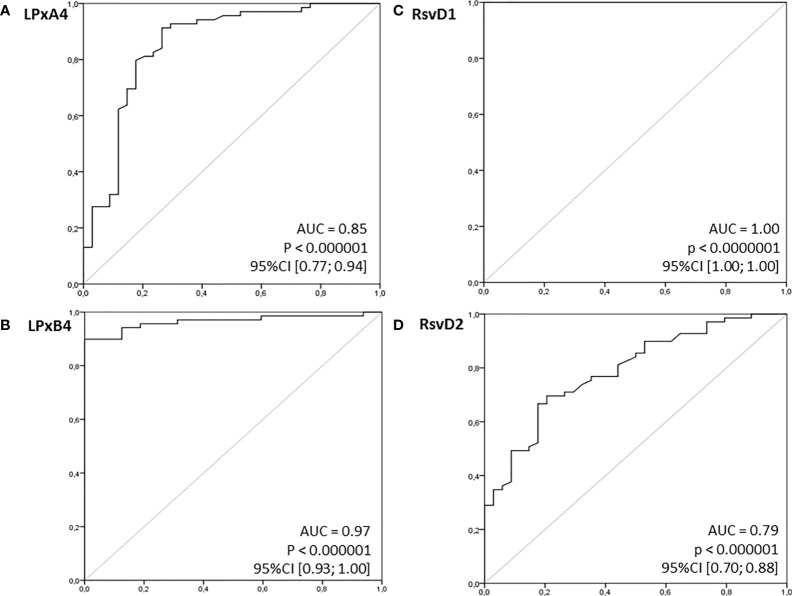
Receiver operating characteristics (ROC) curves for examined immunoresolvents as potential (bio)markers of pancreatic cancer. Calculated sensitivity (y-axis) is plotted against 1-specificity formula (x-axis) for examined immunoresolvents: **(A)** lipoxin A4 (LPxA4), **(B)** lipoxin B4 (LPxB4), **(C)** resolvin D1 (RsvD1) and **(D)** resolvin D2 (RsvD2) as potential indicators of pancreatic cancer. AUC, area under ROC curve; p, level of significance.

In view of the significant differences in the mean concentrations of all immunoresolvents between healthy individuals and patients with pancreatic adenocarcinoma, we performed preliminary analysis to determine whether systemic levels of these substances are of diagnostic value for the detection and/or differentiation of pancreatic cancer in humans. Therefore, we constructed ROC curves for each eicosanoid and calculated the AUC values. Preliminary analysis revealed that systemic levels of all immunoresolvents investigated in this study showed strong diagnostic potential as promising biomarkers of pancreatic adenocarcinoma. AUC values ranged between 0.79 and 1.00 (**Figure 3**). Based on these results, we attempted to determine potential diagnostic cut-off values for levels of the aforementioned immunoresolvents and preliminarily characterized their estimated sensitivity, specificity, and positive and negative predictive values (**Table 3**). We observed that resolvin D1 concentrations showed the most promising results with regard to diagnostic potential (up to 100%). Other newly proposed biomarkers showed approximately 75%–94.1% sensitivity, 65.6%–87.5% specificity, and 82.5%–94.1% and 56.8%–87.5% positive and negative predictive values, respectively.

**Table 3:** Diagnostic value of examined immunoresolvents to discriminate presence of pancreatic adenocarcinoma in our patients.

Parameter	Lipoxin A4	Lipoxin B4	Resolvin D1	Resolvin D2
**Suggested cut-off value**	≥ 893.5 [pg/mL]	≥ 56.4 [pg/mL]	≥ 74.5 [pg/mL]	≥ 39.5 [pg/mL]
**Sensitivity** [%]	91.2	94.1	100.0	76.5
**Specificity** [%]	75.0	87.5	100.0	65.6
**Positive predictive value** [%]	88.5	94.1	100.0	82.5
**Negative predictive value** [%]	80.0	87.5	100.0	56.8

## Discussion

LOX-derived lipoxins and resolvins represent a family of substances that play a major role in successful resolution of inflammation. Therefore, they are also expected to be significant mediators of carcinogenesis, because uncontrolled chronic inflammation is known to be associated with the development of solid malignancies (24–27). However, limited data are available regarding the significance of immunoresolvents in the development of pancreatic adenocarcinoma; few clinical studies have discussed these bioactive lipids in patients with this malignancy. In this study, we investigated a broad panel of immunoresolvents in patients with pancreatic cancer and attempted to determine the clinical associations and diagnostic value, if any, of these substances.

We observed that patients with pancreatic adenocarcinoma showed significantly elevated levels of immunoresolvents, such as lipoxins and resolvins. Our results are consistent with those reported by previous studies, which show significant genetic expression of LOX enzymes in the ductal cells of pancreatic adenocarcinoma and in resected tissue specimens (28–30). Previous studies have reported that only sporadic LOX expression was detected in pancreatic ductal cells in normal human pancreas. In our study, compared with levels in healthy individuals, we observed an approximate 2-fold mean increase in systemic lipoxin levels and specifically a 10-fold or higher increase in systemic resolvin D1 levels in patients with pancreatic cancer. Interestingly, such significant elevations in lipoxin and resolvin levels were observed regardless of the clinical staging of the pancreatic cancer based on the international TNM classification. The mean (elevated) immunoresolvent levels were similar between patients with both early- and advanced (metastatic)-stage disease. Unfortunately, currently, the exact etiology of elevated immunoresolvent levels and the exact molecular consequences of this phenomenon in patients with pancreatic cancer remain unclear. Several experimental studies that have investigated this subject at the molecular level show that immunoresolvents may inhibit pancreatic cancer progression and dissemination. Specifically, research has shown that immunoresolvents may inhibit differentiation of pancreatic stellate cells into cancer-induced fibroblast-like myofibroblasts and “re-program” the tumor stroma, reverse mesenchymal phenotypes of pancreatic cancer cells, and attenuate their invasion and metastasis *via* inhibition of (i) autocrine transforming growth factor β1 signaling, (ii) reactive oxygen species production and, (iii) activity of the extracellular signal regulated kinases that downregulate matrix metalloproteinases (31–33). Based on the findings of these studies, the extent of the spontaneous eicosanoid response in patients with pancreatic cancer remains unclear, and the reason for the lack of a sustained increase in systemic generation of lipoxins and resolvins in advanced-stage pancreatic malignancy remains unexplained. Considering the conclusions drawn from all aforementioned molecular studies, it is reasonable to infer that significantly elevated lipoxin and resolvin levels most likely represent a natural response to pancreatic carcinogenesis in humans, to inhibit uncontrolled inflammation that is a hallmark of pancreatic cancer and additionally accelerates the progression of malignancy. However, further molecular and translational studies are warranted to accurately characterize this phenomenon, particularly focused on the exact mechanisms underlying this process and to verify whether modulation (particularly intensification) of the eicosanoid response could not offer clinical benefit in patients with pancreatic cancer.

In view of the significant differences in peripheral levels of lipoxins and resolvins observed in this study, we preliminarily investigated the diagnostic potential of these eicosanoids as biomarkers of pancreatic adenocarcinoma in humans. ROC curve analysis showed significantly high clinical diagnostic potential of the aforementioned immunoresolvents, particularly of resolvin D1. Therefore, our results support the hypotheses presented by previous studies, which suggest that genetic expression of LOX and/or LOX-derived eicosanoids may be significantly associated with carcinogenesis in addition to being promising biomarkers of various types of solid malignancies (34, 35). We emphasize that our results are preliminary considering the small sample size of our study; further large-scale clinical studies are warranted to validate and characterize the diagnostic potential of the aforementioned immunoresolvents in patients with pancreatic cancer.

In summary, our study highlights the significant alterations in the systemic balance of immunoresolvents, such as lipoxins and resolvins in patients with pancreatic adenocarcinoma and that this finding is unaffected by the clinical TNM stage of the disease. Furthermore, our study is the first to preliminarily measure peripheral levels of lipoxins and resolvins, (particularly resolvin D1), which may potentially serve as novel biomarkers of pancreatic adenocarcinoma in humans.

## Data Availability Statement

The datasets presented in this article are not readily available because data set includes multiple clinical information. Requests to access the datasets should be directed to drannab@wp.pl.

## Ethics Statement

The studies involving human participants were reviewed and approved by the (Bio)Ethics Committee of the Pomeranian Medical University, and study participants provided written informed consent for participation in the study.

## Author Contributions

WB, AD and TS developed and performed the clinical study protocols, as well as evaluated the clinical data. KD and BD developed the laboratory protocols and performed biochemical analyses. WB interpreted results, wrote and revised the article. All authors contributed to the article and approved its final version.

## Conflict of Interest

The authors declare that the research was conducted in the absence of any commercial or financial relationships that could be construed as a potential conflict of interest.

## Publisher’s Note

All claims expressed in this article are solely those of the authors and do not necessarily represent those of their affiliated organizations, or those of the publisher, the editors and the reviewers. Any product that may be evaluated in this article, or claim that may be made by its manufacturer, is not guaranteed or endorsed by the publisher.

## References

[1] Rawla P Sunkara T Gaduputi V. Epidemiology of Pancreatic Cancer: Global Trends, Etiology and Risk Factors. World J Oncol (2019) 10:10–27. doi: 10.14740/wjon1166 30834048 PMC6396775

[2] Ilic M Ilic I. Epidemiology of Pancreatic Cancer. World J Gastroenterol (2016) 22:9694–705. doi: 10.3748/wjg.v22.i44.9694 PMC5124974 27956793

[3] Johnson AM Kleczko EK Nemenoff RA. Eicosanoids in Cancer: New Roles in Immunoregulation. Front Pharmacol (2020) 11:595498. doi: 10.3389/fphar.2020.595498 33364964 PMC7751756

[4] Das UN. "Cell Membrane Theory of Senescence" and the Role of Bioactive Lipids in Aging, and Aging Associated Diseases and Their Therapeutic Implications. Biomolecules (2021) 112:241. doi: 10.3390/biom11020241 PMC7914625 33567774

[5] Hisano Y Hla T. Bioactive Lysolipids in Cancer and Angiogenesis. Pharmacol Ther (2019) 193:91–8. doi: 10.1016/j.pharmthera.2018.07.006 PMC6309747 30048709

[6] Verma G Marella A Shaquiquzzaman M Alam M. Immunoinflammatory Responses in Gastrointestinal Tract Injury and Recovery. Acta Biochim Pol (2013) 60:143–9. doi: 10.18388/abp.2013_1964 23757446

[7] Wang B Wu L Chen J Dong L Chen C Wen Z. Metabolism Pathways of Arachidonic Acids: Mechanisms and Potential Therapeutic Targets. Signal Transduct Target Ther (2021) 6:94. doi: 10.1038/s41392-020-00443-w 33637672 PMC7910446

[8] Calder P. Eicosanoids. Essays Biochem (2020) 64:423–41. doi: 10.1042/EBC20190083 32808658

[9] Panigrahy D Gilligan MM Serhan CN Kashfi K. Resolution of Inflammation: An Organizing Principle in Biology and Medicine. Pharmacol Ther (2021) 107879. doi: 10.1016/j.pharmthera.2021.107879 33915177

[10] Serhan CN Levy BD. Resolvins in Inflammation: Emergence of the Pro-Resolving Superfamily of Mediators. J Clin Invest (2018) 128:2657–69. doi: 10.1172/JCI97943 PMC6025982 29757195

[11] Chandrasekharan JA Sharma-Walia N. Lipoxins: Nature's Way to Resolve Inflammation. J Inflammation Res (2015) 8:181–92. doi: 10.2147/JIR.S90380 PMC4598198 26457057

[12] Greene ER Huang S Serhan CN Panigrahy D. Regulation of Inflammation in Cancer by Eicosanoids. Prostaglandins Other Lipid Mediat (2011) 96:27–36. doi: 10.1016/j.prostaglandins.2011.08.004 21864702 PMC4051344

[13] Lee HN Na HK Surh YS. Resolution of Inflammation as a Novel Chemopreventive Strategy. Semin Immunopathol (2013) 35:151–61. doi: 10.1007/s00281-013-0363-y 23370700

[14] Fishbein A Hammock BD Serhan CN Panigrahy D. Carcinogenesis: Failure of Resolution of Inflammation? Pharmacol Ther (2021) 218:107670. doi: 10.1016/j.pharmthera.2020.107670 32891711 PMC7470770

[15] de-Brito NM da-Costa HC Simoes RL Barja-Fidalgo C. Lipoxin-Induced Phenotypic Changes in CD115 + LY6C Hi Monocytes TAM Precursors Inhibits Tumor Development. Front Oncol (2019) 9:540. doi: 10.3389/fonc.2019.00540 31275860 PMC6593314

[16] Khophai S Thanee M Techasen A Namwat N Klanrit P Titapun A. Zileuton Suppresses Cholangiocarcinoma Cell Proliferation and Migration Through Inhibition of the Akt Signaling Pathway. Onco Targets Ther (2018) 11:7019–29. doi: 10.2147/OTT.S178942 PMC6198876 30410359

[17] Claria J Lee MH Serhan CN. Aspirin-Triggered Lipoxins (15-Epi-LX) Are Generated by the Human Lung Adenocarcinoma Cell Line (A549)-Neutrophil Interactions and Are Potent Inhibitors of Cell Proliferation. Mol Med (1996) 2:583–96. doi: 10.1007/BF03401642 PMC2230193 8898374

[18] Gilligan MM Gartung AA Sulciner ML Norris PC Sukhatme VP Bielenberg DR. Aspirin-Triggered Proresolving Mediators Stimulate Resolution in Cancer. Proc Natl Acad Sci USA (2019) 116:6292–7. doi: 10.1073/pnas.1804000116 PMC6442621 30862734

[19] Deskur A Salata D Budkowska M Dolegowska B Starzynska T Blogowski W. Selected Hemostatic Parameters in Patients With Pancreatic Tumors. Am J Transl Res (2014) 6:768–76. PMC4297344 25628787

[20] Blogowski W Dolegowska K Deskur A Dolegowska B Starzynska T. An Attempt to Evaluate Selected Aspects of "Bone-Fat Axis" Function in Healthy Individuals and Patients With Pancreatic Cancer. Medicine (2015) 94:e1303. doi: 10.1097/MD.0000000000001303 26266370 PMC4616689

[21] Bodnarczuk T Deskur A Dolegowska K Dolegowska B Starzynska T Blogowski W. Hydroxyeicosatetraenoic Acids in Patients With Pancreatic Cancer: A Preliminary Report. Am J Cancer Res (2018) 8:1865–72. PMC6176181 30323978

[22] Madej-Michniewicz A Budkowska M Salata D Dolegowska B Starzynska T Blogowski W. Evaluation of Selected Interleukins in Patients With Different Gastric Neoplasms: A Preliminary Report. Sci Rep (2015) 5:14382. doi: 10.1038/srep14382 26486258 PMC4613562

[23] Blogowski W Madej-Michniewicz A Marczuk N Dolegowska B Starzynska T. Interleukins 17 and 23 in Patients With Gastric Neoplasms. Sci Rep (2016) 6:37451. doi: 10.1038/srep37451 27869179 PMC5116626

[24] Zhang Q Zhu B Li Y. Resolution of Cancer-Promoting Inflammation: A New Approach for Anticancer Therapy. Front Immunol (2017) 8:71. doi: 10.3389/fimmu.2017.00071 28210259 PMC5288347

[25] Zhang T Hao H Zhou XY. The Role of Lipoxin in Regulating Tumor Immune Microenvironments. Prostaglandins Other Lipid Mediat (2019) 144:106341. doi: 10.1016/j.prostaglandins.2019.106341 31152809

[26] Tian R Zuo X Jaoude J Mao F Colby J Shureiqi I. ALOX15 as a Suppressor of Inflammation and Cancer: Lost in the Link. Prostaglandins Other Lipid Mediat (2017) 132:77–83. doi: 10.1016/j.prostaglandins.2017.01.002 28089732 PMC5509529

[27] Janakiram NB Rao CV. Role of Lipoxins and Resolvins as Anti-Inflammatory and Proresolving Mediators in Colon Cancer. Curr Mol Med (2009) 9:565–79. doi: 10.2174/156652409788488748 19601807

[28] Knab LM Grippo PJ Bentrem DJ. Involvement of Eicosanoids in the Pathogenesis of Pancreatic Cancer: The Roles of Cyclooxygenase-2 and 5-Lipoxygenase. World J Gastroenterol (2014) 20:10729–39. doi: 10.3748/wjg.v20.i31.10729 PMC4138453 25152576

[29] Zhou GX Ding XL Wu SB Zhang HF Cao W Qu LS. Inhibition of 5-Lipoxygenase Triggers Apoptosis in Pancreatic Cancer Cells. Oncol Rep (2015) 33:661–8. doi: 10.3892/or.2014.3650 25483364

[30] Hennig R Ding XZ Tong WG Schneider MB Standop J Friess H. 5-Lipoxygenase and Leukotriene B(4) Receptor Are Expressed in Human Pancreatic Cancers But Not in Pancreatic Ducts in Normal Tissue. Am J Pathol (2002) 161:421–8. doi: 10.1016/S0002-9440(10)64198-3 PMC1850753 12163367

[31] Schnittert J Heinrich MA Kuninty PR Storm G Prakash J. Reprogramming Tumor Stroma Using an Endogenous Lipid Lipoxin A4 to Treat Pancreatic Cancer. Cancer Lett (2018) 420:247–58. doi: 10.1016/j.canlet.2018.01.072 29408203

[32] Zong L Chen K Jiang Z Chen X Sun L Ma J. Lipoxin A4 Reverses Mesenchymal Phenotypes to Attenuate Invasion and Metastasis *via* the Inhibition of Autocrine TGF-β1 Signaling in Pancreatic Cancer. J Exp Clin Cancer Res (2017) 36:181. doi: 10.1186/s13046-017-0655-5 29228980 PMC5725800

[33] Zong L Li J Chen X Chen K Li W Li X. Lipoxin A4 Attenuates Cell Invasion by Inhibiting ROS/ERK/MMP Pathway in Pancreatic Cancer. Oxid Med Cell Longev (2016) 2016:6815727. doi: 10.1155/2016/6815727 26649143 PMC4663743

[34] Chen A Zhang Y Sun D Xu S Guo Y Wang X. Investigation of the Content Differences of Arachidonic Acid Metabolites in a Mouse Model of Breast Cancer by Using LC-MS/MS. J Pharm BioMed Anal (2021) 194:113763. doi: 10.1016/j.jpba.2020.113763 33279296

[35] Ruan GT Gong YZ Zhu LC Gao F Liao XW Wang XK. The Perspective of Diagnostic and Prognostic Values of Lipoxygenases mRNA Expression in Colon Adenocarcinoma. Onco Targets Ther (2020) 13:9389–405. doi: 10.2147/OTT.S251965 PMC7520158 33061426

